# Kinematic adaptions to induced short-term pelvic limb lameness in trotting dogs

**DOI:** 10.1186/s12917-018-1484-2

**Published:** 2018-06-13

**Authors:** Birte Goldner, Stefanie Fischer, Ingo Nolte, Nadja Schilling

**Affiliations:** 1 Foundation, Small Animal Clinic, University of Veterinary Medicine Hannover, Hannover, Germany; 2 Institute of Systematic Zoology and Evolutionary Biology, Friedrich-Schiller-University, Jena, Germany

**Keywords:** Hindlimb lameness, Kinematics, Compensatory mechanism, Angular excursion

## Abstract

**Background:**

Lameness due to paw injuries is common in the clinical practice. Although many studies investigated gait adaptations to diseases or injuries, mainly of the hip and knee, our understanding of the biomechanical coping mechanisms that lame dogs utilize is limited. Therefore, this study evaluated the kinematic changes associated with an induced, load-bearing pelvic limb lameness in healthy dogs trotting on a treadmill. Kinematic analysis included spatio-temporal comparisons of limb, joint and segment angles of all limbs. Key parameters compared between sound and lame conditions were: angles at touch-down and lift-off, minimum and maximum joint angles and range of motion.

**Results:**

Significant differences were identified in each limb during both stance and swing phases. The most pronounced differences concerned the affected pelvic limb, followed by the contralateral pelvic limb, the contralateral thoracic limb and, to the least degree, the ipsilateral thoracic limb. The affected limb was retracted more, while the contralateral limb was protracted more, consistent with this limb bearing more body weight in lame dogs.

**Conclusions:**

Kinematic adaptations involved almost all segment and joint angles in the pelvic limbs, while they exclusively concerned distal parts of the thoracic limbs. Comparisons with tripedal locomotion reveal several striking similarities, implying that dogs use similar principles to cope with a partial or a total loss in limb function. Because kinematic alterations occurred in all limbs and not just the affected one, all limbs should be included in routine follow-ups and be part of the diagnostic and therapeutic care of canine patients.

**Electronic supplementary material:**

The online version of this article (10.1186/s12917-018-1484-2) contains supplementary material, which is available to authorized users.

## Background

Canines with paw injuries are common patients in the clinical routine. They have stepped into things, have lacerations, fractured toes or injured their claws. Medical treatment may be easy, but, depending on the extent of the injury, the patients usually unload the affected limb to prevent further damage and assuage the pain. In result, their gait displays alterations (e.g. lameness) and the sound limbs must compensate for the reduction in one limb’s function. Similarly, dogs with orthopaedic pathologies such as hip dysplasia or cranial crucial ligament rupture have to biomechanically circumvent the reduction in limb function and consequently adjust their gait. Although gait analysis has become an important diagnostic tool over the last decades, the specific adaptations to lameness and their consequences for the locomotor system are not well understood.

Many biomechanical analyses of pelvic limb lameness have been carried out in dogs to determine alterations for example in the external and internal forces (e.g. [1–10]) or the muscle activation patterns (e.g. [11, 12]). Other studies focussed on kinematic adaptations because 1) kinematic analysis is an important diagnostic tool [13, 14] and provides insight into compensatory mechanisms additionally to kinetic or electromyographic details [13, 15, 16], 2) different orthopaedic conditions have been suggested to exhibit specific kinematic signatures that potentially bear diagnostic value [13, 17, 18] and 3) kinematic deviations may be seen before changes in the external forces can be detected or the dogs show clinical signs [13, 19, 20]. Moreover, some studies suggested that gait adaptations might depend on whether lameness is caused by a proximal vs. a distal dysfunction [21, 22]. That is, the displayed gait alterations might indicate whether the hip, knee, ankle or the paw is affected.

Because of their prevalence [23], kinematic investigations focused on lameness due to hip dysplasia and hip osteoarthritis (e.g. [11, 17–20, 24]) or cranial cruciate ligament rupture (e.g. [9, 25–31]). In contrast to this relatively large body of information, kinematic alterations due to injuries or dysfunctions of the paw have not been studied. Furthermore, some of these studies considered the pelvic limbs only disregarding the thoracic limbs (e.g. [17–19]). Others assumed the sound limb could serve as a control [14] or looked at the complete locomotor cycle without distinguishing stance and swing phases (e.g. [2, 32]). However, lameness of one limb has been shown to affect all limbs and both, stance and swing phases [16, 20, 26].

To gain insight into the kinematic changes resulting from pelvic limb lameness, this study determined limb, segment and joint angles for all four limbs in 8 dogs trotting on a treadmill. To allow for the direct comparison of the data for the sound and lame conditions under relatively controlled conditions, thus reducing the number of factors potentially introducing variability into the results (e.g. cause, severity, duration of lameness), this study collected kinematic data from the same individuals before and after a moderate, load-bearing, short-term lameness was induced in the right pelvic limb. The lameness model used herein has been previously shown to be of value in the analysis of gait alterations due to losses of limb function (e.g. [10, 33]). By comparing our results with previous findings, we aimed at, firstly, identifying similarities and differences in the kinematic adaptations to pelvic limb lameness caused by a more proximal vs. a more distal dysfunction (i.e. hip or knee vs. paw). Secondly, to test whether dogs use fundamentally similar strategies to unload a pelvic limb, this study compared the results from this study (ca. 33% less peak vertical force; [10]) with previous observations of dogs ambulating tripedally (100% unloading; [16]).

Note, that results of adaptations in vertical force and temporal gait parameters are presented in companion study [10].

## Methods

### Animals

All dogs belonged to the Beagle population of the Small Animal Clinic of the University of Veterinary Medicine Hannover (Germany). The seven males and one female had a body mass of 15.1 ± 1.2 kg (mean ± standard deviation, SD) and were 4 ± 1 years old. Inclusion criteria were absence of orthopaedic abnormalities and lameness verified by clinical examination and evaluation of the load-bearing characteristics of the limbs [10]. The experiments were carried out in accordance with the German Animal Welfare guidelines and approved by the Ethical committee of the State of Lower Saxony, Germany (12/0717; 2012–12-04).

### Study design

To allow for the direct comparison with previous results, the same experimental protocol was used [10, 12, 16]. In summary, the dogs were habituated to trotting on a horizontal four-belt treadmill with force plates mounted underneath each belt; thus, allowing for the synchronous recording of single limb ground reaction forces (Modell 4060–08, Bertec Corporation, OH, USA; sampling rate: 1000 Hz, force threshold: 13 N). When the dogs trotted smoothly and comfortably, data collection started. Each recording session started with a 5 min warm up before the dog was instrumented (see below) and control data (i.e. unimpeded trotting) obtained. For each dog, ca. 15 trials were recorded each one lasting for about 20–30 s.. After a break of at least 15 min, a reversible lameness was induced on the right pelvic limb (i.e., reference limb, ipsilateral side) by taping a small sphere under the paw (for details, see [33]). Data collection was then repeated and, on average, 11 trials were recorded for the lame condition. During both, control and lameness data collections, all dogs trotted at the same speed: 1.4 m/s. The same experienced experimenter (SF) handled all dogs to ensure unrestrained and steady state locomotion.

### Data collection

Twenty-two retro-reflective markers (diameter: 10 mm) were placed above defined anatomical landmarks using adhesive tape (for details, see Fig. 1 in [16]). Six infrared high-speed cameras (MX 3+, Vicon Motion Systems Ltd. Oxford, UK) tracked the marker positions three-dimensionally. A high-speed video camera recorded the dogs from the lateral perspective (Basler Pilot, PiA640-210gc, Ahrensburg, Germany; sampling rate: 100 Hz). Prior to the data collection, the cameras were calibrated using an L-shaped calibration device (Vicon Motion Systems Ltd., Oxford, UK). The signals of the infrared cameras, the high-speed video camera and the force plates were recorded synchronously in Vicon Nexus.

**Fig. 1:**
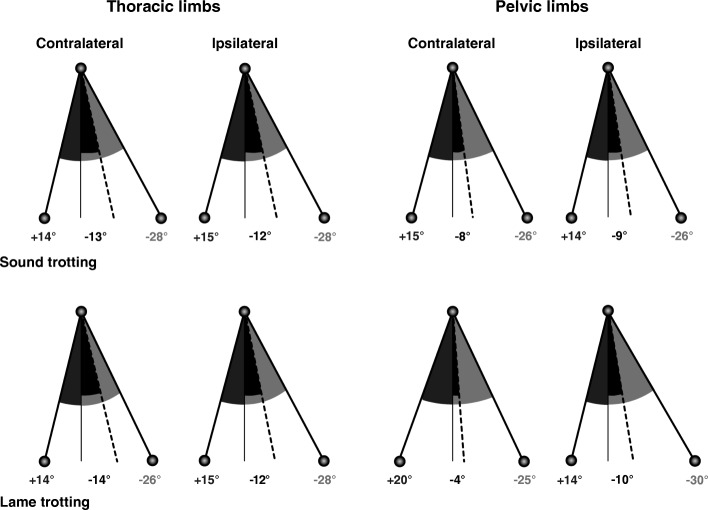
Limb angles during sound and lame trotting. Comparison of thoracic and pelvic limb angles at touch-down (dark grey), lift-off (light grey) and mid-stance (black, dashed line) relative to the vertical during sound and lame trotting. Numbers below each stick-figure represent the means for all dogs (see Fig. 1 in 16 for further explanation and Appendix: Supplementary Tables S1 and S2 for details). Note the greater retraction of the affected (i.e. ipsilateral) and the greater protraction of the contralateral pelvic limbs

### Data analysis

Kinematic data analyses followed previously established protocols [16]. In short, 10 consecutive strides per dog and condition were selected for analysis. The vertical force traces served to define touch-down and lift-off for each limb. Using customized kinematic models, the markers were labelled in Vicon Nexus and limb, segment and joint angles determined as defined previously (see Fig. 1 in [16]). Note that all data are two-dimensional (i.e. the angles were projected onto the sagittal plane). Before export to Microsoft Excel 2003, the data were time-normalized to the same stance and swing phase durations to facilitate the comparison of the movements with reference to the footfall events (for details, see [10, 34]). In result, each stride phase covered 50% of the locomotor cycle.

Additionally to segment and joint angles at touch-down and lift-off, minima, maxima and ranges of motion (ROM) during stance and swing phases were compared between conditions. Overall limb excursion was assessed via the limb’s angle at touch-down and lift-off. The angle between the vertical and the line connecting the limb’s fulcrum with the most distal marker at mid-stance was used to determine limb angle at mid-stance. Furthermore, note that this study focused on angular rotations (i.e. ante- and retroversion or cranial and caudal rotations, respectively) and does not consider translatory motions (i.e. pro- and retraction or cranial and caudal translations). To distinguish limb from segment movements, we use protraction and retraction for a limb’s and anteversion and retroversion for a segment’s cranial and caudal rotation, respectively.

### Statistical analysis

Because of the small sample size (*N* = 8), non-parametric Wilcoxon-signed rank tests for paired observations and accounting for comparison-wise error rate were used to detect kinematic differences between sound and lame conditions (significant at *P* < 0.05). All tests were performed in GraphPad Prism 4. To further specify significant differences, we compared the angular excursions using a time-series comparison (i.e. bin-by-bin analysis after [34]). In the following, we will present the results from the lame condition relative to the control values.

## Results

**Fig. 2:**
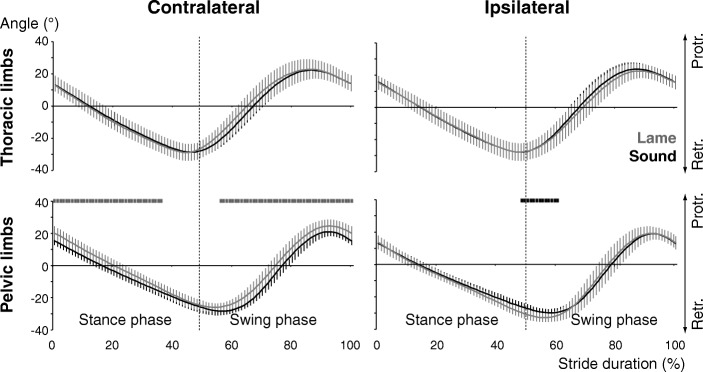
Angular excursions during sound and lame trotting. Stride-phase normalized angular excursion of the thoracic (top) und pelvic (bottom) limbs. The traces represent the mean and standard deviation (error bars) of all dogs during sound (black) and lame (grey) trotting. Bars above each graph indicate significant differences based on the bin-by-bin analysis with the respective colour indicating the significantly greater value. Labels on the right indicate increasing pro- or retraction of the limb (i.e. Protr. and Retr., respectively)

**Fig. 3:**
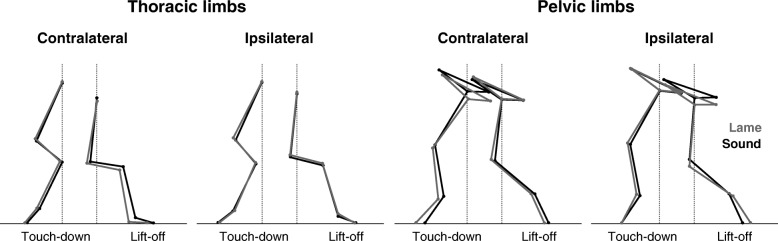
Thoracic and pelvic limb positions during sound and lame trotting. Stick-figures of the thoracic and pelvic limbs illustrating limb positions at touch-down and lift-off during sound (black) and lame trotting (grey) when averaged for all dogs

Trotting with a short-term, load-bearing lameness was associated with significant kinematic changes in all four limbs that affected both stance and swing phases (except the contralateral tarsal joint, which displayed stance phase changes only; see [Additional files 1 and 2]). Compared with sound trotting, the affected pelvic limb showed the greatest number of kinematic differences (30 out of 59 parameters analysed per limb) followed by the contralateral pelvic (*N* = 25), the contralateral thoracic (*N* = 14) and the ipsilateral thoracic limb (*N* = 4). While more changes were associated with the stance than the swing phase in the pelvic limbs (ipsilateral: 13–12; contralateral: 12–8), stance and swing phase changes were equal in the contralateral thoracic limb (5–5) and more swing than stance changes were detected in the ipsilateral thoracic limb (2–1). Averaging the significant changes of the segment and joint angles shows that the mean degree of angular change was greatest in the contralateral and smallest in the ipsilateral thoracic limb (8.4 ± 3.4° vs. 3.3 ± 3.7°). In the pelvic limbs, the affected side showed a barely notable greater mean angular change than the sound one (5.4 ± 3.5° vs. 4.7 ± 3.4°). Regarding overall limb posture, the contralateral pelvic limb was protracted more resulting in a greater angle at touch-down, a more vertical position at mid-stance and a greater stance angular velocity (Figs. 1 and 2). The ipsilateral pelvic limb was retracted more connected with a greater angle at lift-off. Among of the more noticeable differences was the vertical lowering of the hip and the greater anteversion of the pelvis around the contralateral limb’s touch-down and the ipsilateral limb’s lift-off (Fig. 3).

### Thoracic limbs

**Fig. 4:**
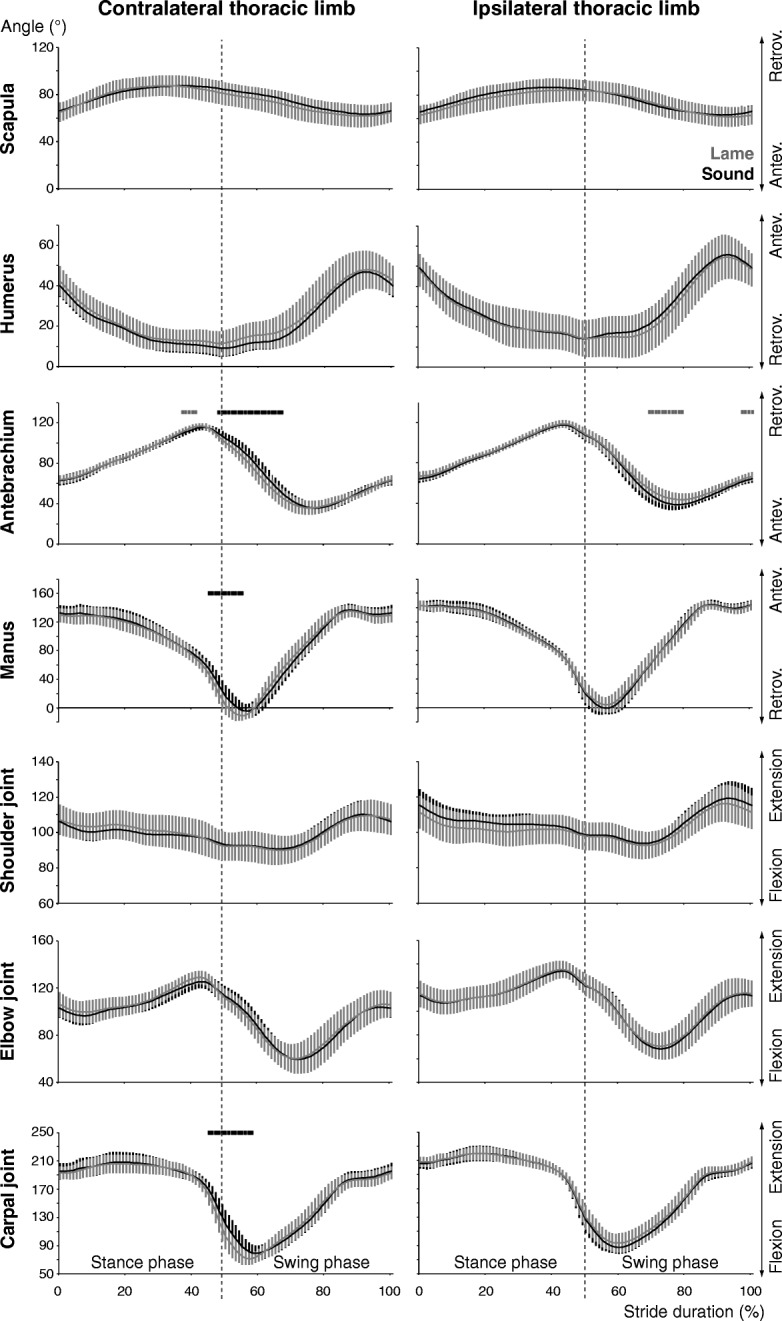
Thoracic limb segment and joint angular excursions. Stride-phase normalized angular excursions of the segment and joint angles of the thoracic limbs (further details as in Fig. 3). Labels on the right indicate increasing ante-or retroversion for the segments (i.e. Antev. and Retrov., respectively) and flexion and extension for the joints

In both thoracic limbs, the significant kinematic differences concerned distal limb parts only. Specifically, the contralateral carpal joint was flexed more during late stance and early swing phase due to a reduced retroversion of the antebrachium and anteversion of the manus (Fig. 4). The greater flexion of the carpal joint was associated with a smaller angle at lift-off and a reduced stance minimum, which resulted in an increased ROM. The greater carpal flexion lasted into swing associated with a smaller minimum see (Additional file 1). In the ipsilateral limb, the antebrachium was retroverted more at touch-down leading to a greater stance minimum. During swing, its reduced anteversion resulted in a smaller ROM (Fig. 4).

### Pelvic limbs

**Fig. 5:**
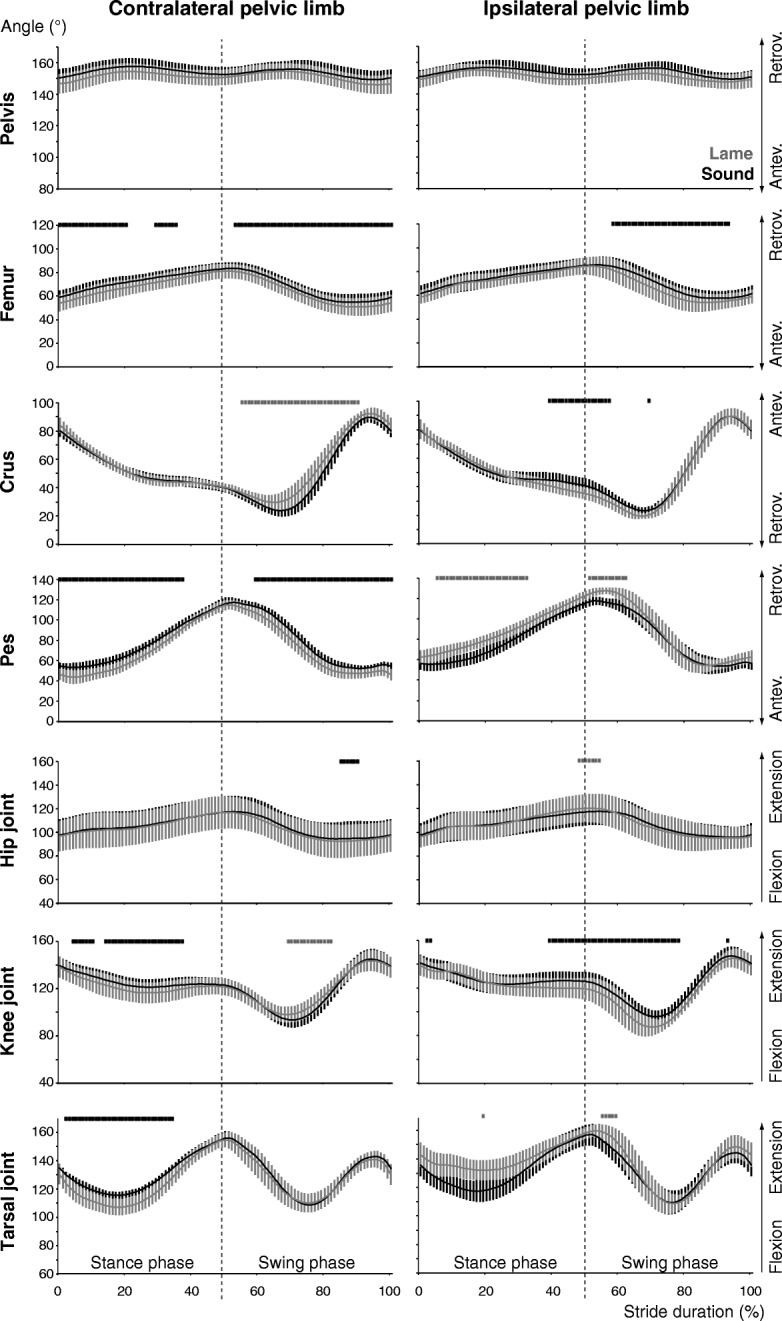
Pelvic limb segment and joint angular excursions. Stride-phase normalized angular excursions of the segment and joint angles of the pelvic limbs (further details as in Fig. 4)

Except the hip joint, kinematic changes were observed for all segment and joint angles of the contralateral pelvic limb see (Additional file 2). Due to the greater anteversion of the pelvis, femur, crus and pes were also anteverted more throughout most of the locomotor cycle (Fig. 5). Only around lift-off, segment and joint angles were comparable to the sound condition (Fig. 3). During most of the stance phase, knee and tarsal joints were flexed more. The more extended knee around mid-swing was associated with a less retroverted crus. The ipsilateral hip joint was extended more around lift-off due to greater pelvic anteversion. Because of the greater retroversion of the crus, the knee joint was flexed more from mid-stance till mid-swing. For most of the stance phase, the tarsal joint was extended more, which resulted from an overall more retroverted pes. Finally, the femur displayed less retroversion during most of the swing phase.

## Discussion

Aiming at a better understanding of the compensatory mechanisms that dogs utilize to cope with a partial loss of a pelvic limb’s function, this study analysed the kinematic changes that were associated with an induced, load-bearing lameness. Several kinematic studies previously investigated the effects of knee or hip dysfunctions on the gait pattern, e.g. to evaluate treatment options or compare benefits of various operation techniques (see below). The current study adds information to this database, because it addresses kinematic changes associated with induced paw lameness.

Different orthopaedic conditions were suggested to exhibit specific kinematic signatures and therefore potentially have diagnostic value [13, 17, 18]. If so, we would expect certain dysfunctions to be associated with unique kinematic alterations, which then may help to identify the affected limb’s part. The comparison among previously published data implies that no unique kinematic identifiers exist for hip dysfunction because the kinematic changes clearly differed between the studies ([17, 24]; Table 1). In contrast, studies analysing dysfunctions of the knee uniformly observed greater hip extension during late swing, at touch-down and early stance [9, 26, 27]. Although the database is still small, an increased stance range of motion of the hip joint may point to a dysfunction of the paw.

**Table 1:**
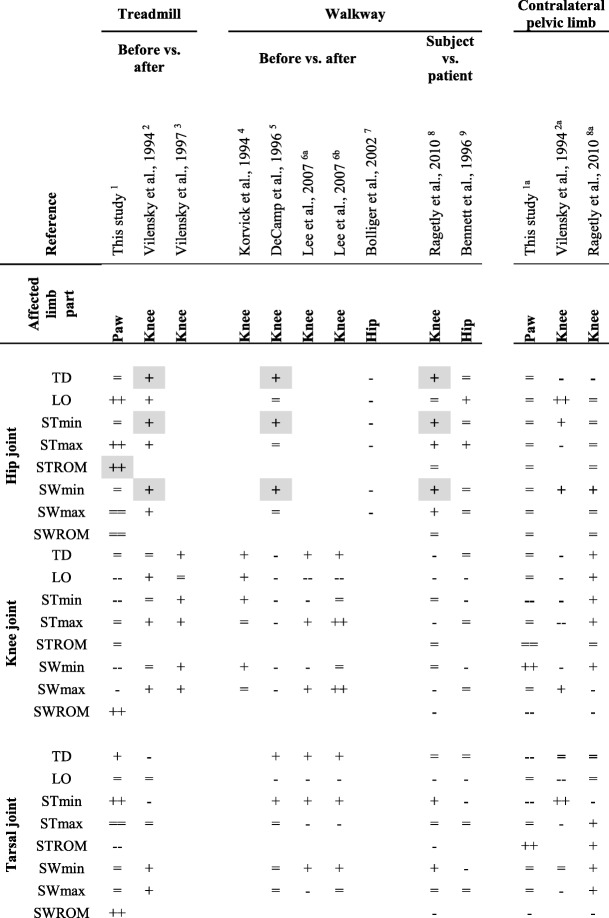
Kinematic adaptations to lameness observed in previous and this study

^1^Lamness induction, before vs. after lameness induction, right paw

^2^Transection of the cranial cruciate ligament (CCL), pre-OP vs. 1 week post-OP, left knee

^3^Deafferentiation and CCL-transection, healthy pre-OP vs. 1 week post-CCL-transection, left knee

^4^CCL-transection plus bone implants for kinematic analysis, pre-OP vs. 7 weeks post-OP, left knee

^5^CCL-transection, pre-OP vs. 4 weeks post-OP, right knee

^6a^Tibial plateau levelling osteotomy or

^6b^Cranial tibial wedge osteotomy after CCL-transection, 2 weeks pre-OP vs. 8 weeks post-OP, left knee

^7^Hip dysplasia, gold bead and placebo implantation before vs. 4 weeks after, more affected side

^8^CCL-transection, clinically healthy vs. CCL dogs, unilaterally affected

^9^Hip dysplasia, clinically healthy vs. HD dogs, more affected side

Comparison of the kinematic changes observed in the joint angles of the affected pelvic limb (left columns) as well as the contralateral pelvic limb (right columns) in this vs. previous studies. The dogs trotted either on a treadmill or along a walkway. Study designs included before vs. after and subject vs. patient comparisons. Lameness was caused by dysfunctions of the ipsilateral paw, knee or hip (see footnotes for details). Kinematic values are: angle at touch-down (TD) and lift-off (LO) as well as minimum (min), maximum (max) and amplitude (i.e. range of motion, ROM) during stance (ST) and swing (SW) phases. Kinematic parameters in the respective comparisons increased (+), decreased (−) or were unchanged (=). Note that only kinematic changes ≥3° were considered an in- or decrease for this comparison. The threshold was based on the mean intraindividual variability observed in the current study (i.e. mSDs averaged across all limbs and conditions; Appendix: Supplementary Tabs. S1, S2). Double-pluses or -minuses indicate that the observed kinematic differences were significant in the respective study. Double-equals indicate that a significant change was observed but below the threshold used herein. No sign indicates that this parameter was not evaluated in the respective study. Similarities in the kinematic changes among studies looking at the dysfunctions of the paw, knee or hip, which potentially bear diagnostic value are bold and highlighted in grey. Also, note that some studies provided graphical representations of the data rather than tables ^(2–7, 9)^, thus hindering a more exact comparison of the kinematic information

Comparing the kinematic changes due knee vs. paw dysfunctions, agreement between results ranged from none ([28] vs. this study) to about half of the parameters analysed ([27] vs. this study). The study most comparable to the current one regarding experimental design showed similar kinematic changes in one third of the parameters [26]. Interestingly, our results were largely in accordance with the kinematic changes observed due to hip dysplasia, allowing for the fact, that comparable database is still small ([17]; Table 1).

Previous studies including the contralateral pelvic limb reported greater hip flexion during swing and at touch-down after CCL-transection [9, 26]. The only kinematic change that exclusively characterized trotting with pelvic limb lameness due to paw dysfunction was a significantly smaller tarsal angle at touch-down.

### Lameness vs. Tripedal

Invited by the observations that dogs shift their centre of body mass, alter their external forces, adapt their temporal gait parameters and change the recruitment patterns of their limb and back extensor muscles in strikingly similar ways when coping with a partial or total loss of limb function [10, 12, 33, 35–37], we hypothesized that dogs may utilize similar principles to biomechanically compensate for reduced or lost limb functions. If that were true, we would expect the gait alterations observed when limb load was reduced ([10]; this study) to be suggestive of the gait changes observed when limb load was zero [16, 36]. The following similarities in the specific gait adaptations to trotting with pelvic limb lameness and trotting on three limbs support our hypothesis.

1) All sound limbs showed kinematic changes in adaptation to trotting with induced pelvic limb lameness (L) and trotting tripedally (T). 2) In both conditions, the limb with the most numerous changes was the contralateral pelvic limb, followed by the contralateral thoracic and the ipsilateral thoracic limbs (L: 25–14-4; T: 34–25-14; Table 2). 3) Kinematic alterations concerned both the stance and the swing phase of the sound limbs. Thereby, the stance phase was affected more often than the swing phase in the contralateral pelvic limb, while more swing than stance phase changes were observed in the ipsilateral thoracic limb (stance-swing: L: 12–8 vs. 1–2; T: 14–12 vs. 2–10). 4) Body weight redistribution was similar during lame and tripedal trotting; that is, the load increased the most in the contralateral pelvic limb, less in the contralateral thoracic limb, while no change was observed in the ipsilateral thoracic limb. 5) The contralateral pelvic limb was protracted more, consistent with this limb supporting a greater proportion of the body weight. 6) The pelvis was anteverted more, facilitating the increased protraction of the contralateral pelvic limb. 7) Relative stance duration increased the most in the contralateral pelvic limb, less but still significantly in the contralateral thoracic limb, while the ipsilateral thoracic limb showed either no change (L) or an increase (T). 8) To maintain overall limb cycle duration while accounting for the relative longer time a limb is on the ground, this limb has to move more quickly during swing. Accordingly, limb angular velocity changes affected primarily the swing phases in both conditions.

**Table 2:**
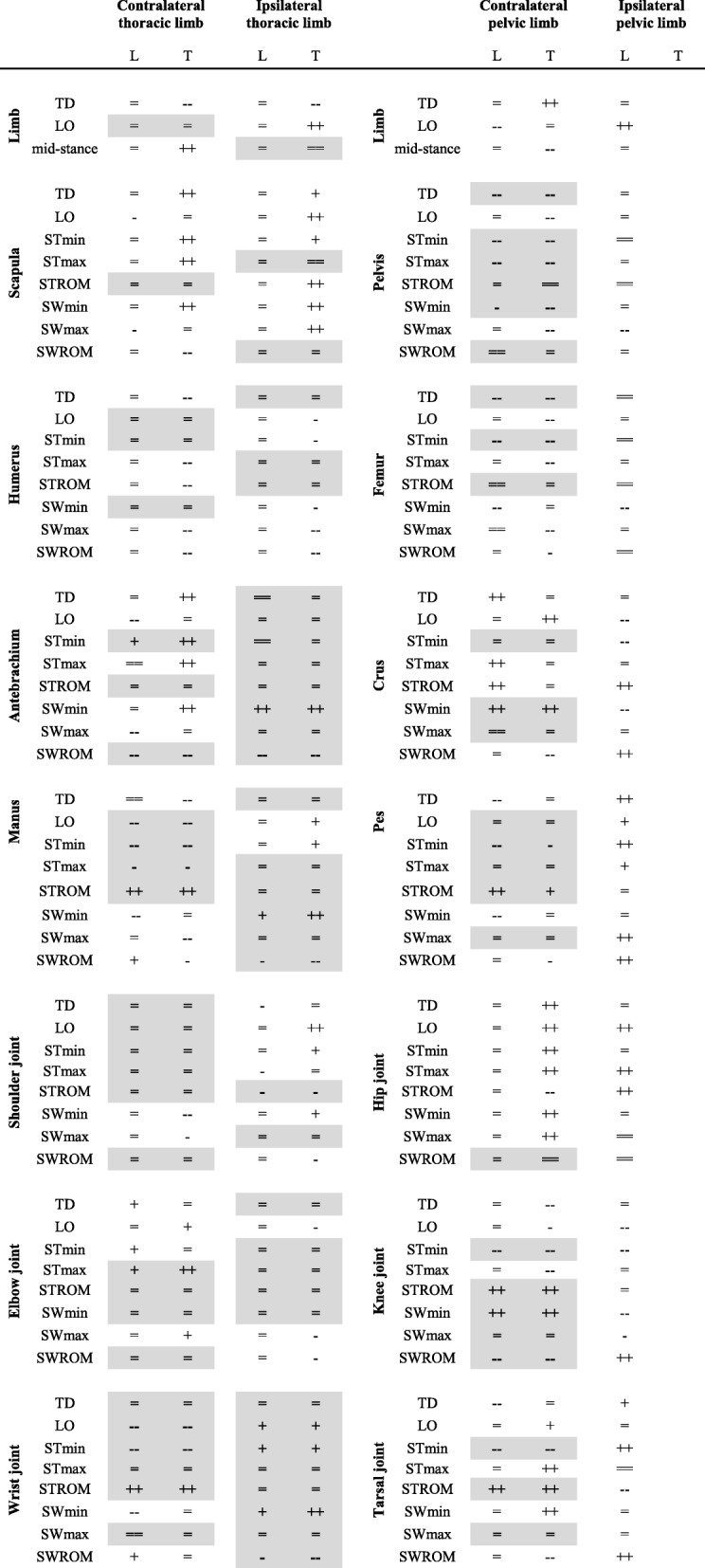
Kinematic comparison between lame and tripedal trotting

Comparison of the kinematic changes observed in this study (L: load-bearing pelvic limb lameness) vs. our previous study on tripedal locomotion (T; [16]). For further details and abbreviations, see Table 1. Similarities in the kinematic changes between a partial and a total loss in pelvic limb function are bold and highlighted in grey

Despite these similarities, some differences existed in the way dogs adjust their kinematic parameters to trotting with lameness vs. tripedally because a reduction in limb function is not simply a milder form of a total loss. 1) Both thoracic limbs were retracted more when the dogs trotted on three legs in order to shift the centre of body mass cranially and unload the remaining pelvic limb. In this study, thoracic limb posture was unchanged, likely because the degree to which the centre of body mass had to be shifted was substantially less. 2) When lame dogs trot, the affected pelvic limb was retracted more at lift-off, connected with an increased relative stance duration and a significantly later lift-off [10]. In result, the temporal overlap between the ground contacts of the diagonal limbs was increased, while the gap between the stance phases of the pelvic limbs was decreased, possibly to increase the time during which the limbs can exert forces. 3) Both, the degree of angular changes as well as whether stance or swing phases were affected more often differed between conditions for the contralateral thoracic limb. Stance adaptations were as numerous as swing adaptations during lame trotting, while more adaptations occurred during stance than swing in tripedally trotting dogs (5–5 vs. 11–8).

Taken together, the kinematic alterations observed in this and our companion study show, first, that dogs involve the limb diagonal to the affected limb (i.e. the contralateral thoracic limb) because diagonal limbs set down and exert forces in unison during trotting [38]; thereby, allowing one limb’s functional loss to be compensated by the other limb of the supporting pair [36]. Second, that dogs, engage the limb opposite to the affected one (i.e. the contralateral pelvic limb, that seems to give a push to lift the affected limb) when coping with the functional loss, the more intensive the more substantial the loss is. This, in turn, entails adaptations of the diagonal limb of the unaffected limb pair (i.e. the ipsilateral thoracic limb). Although the current data basis is small and further studies are necessary to fill the data gap between partial and total functional losses, we are confident that the observed similarities and differences in the kinematic changes result first and foremost from the different degrees of unloading. In both studies, we used the same experimental design regarding the dogs’ locomotor speed, breed, age, body size, health status and gait as well as regarding the equipment, marker placement and data analyses and therefore, were able to minimize the sources that often introduce variability (for detailed discussion, see [16]).

## Conclusions

The results of this study indicate substantial kinematic changes concerning all four limbs during both stance und swing phases. The most affected limb was the ipsilateral pelvic limb, followed by the contralateral pelvic limb, the contralateral thoracic and the ipsilateral thoracic limb. Because kinematic changes were identified in all limbs even if load reduction is only a third, all limbs should be included in routine follow-ups and not just the limb obviously being affected by a disease or an injury. The striking similarities in the gait adaptations including now kinematic parameters supports our hypothesis that dogs manage a partial and a total loss of a limb’s function in similar manners, notwithstanding some specific adaptations depending on the degree of functional loss.

## Additional files

Additional file 1: Kinematic results for the thoracic limbs. Mean ± standard deviation (Mean ± SD in °) of the limb, segment and joint angles for all dogs. Kinematic values for the limbs are: angle at touch-down (TD), lift-off (LO) and mid-stance (mid-stance). Kinematic values for the segments and joints are: angle at touch-down (TD) and lift-off (LO) as well as minimum (min), maximum (max) and amplitude (i.e. range of motion, ROM) during stance (ST) and swing (SW) phases. Mean SDs (mSD in °; i.e. SDs from the 10 strides per dog averaged for all dogs) illustrate the relatively low intraindividual variation compared with the interindividual variation (SD of Mean ± SD) and particularly compared with the angular difference between sound and lame trotting (Diff Mean ± SD in °). Note that this mean Diff was calculated by, first, subtracting the lame from the sound values per dog and, second, averaging these angular differences for all dogs (i.e. mean Diff represents the angular changes associated with lame locomotion). Positive Diff values indicate that the angle was greater during sound than lame trotting; negative values indicate the reverse. Significant differences between sound and lame trotting for each limb (I) as well as significant differences between the angular differences of the two limbs (II) at: * *P* < 0.05, ** *P* < 0.01, *** *P* < 0.001. For definition of angles, see Fig. 1 in [16]. (DOC 349 kb)

Additional file 2: Kinematic results for the pelvic limbs. For further explanation, see Additional file 1. (DOC 332 kb)

## Abbrevations

Antevers: Anteversion
CCL: Cranial cruciate ligament
Diff: Difference
HD: Hip dysplasia
L: Trotting with induces pelvic limb lameness
LO: Lift-off
mSD: Mean standard deviation
Protr: Protraction
Retr: Retraction
Retrovers: Retroversion
ROM: Range of motion
SD: Standard deviation
STmax: Maximum of stance phase
STmin: Minimum of stance phase
STROM: Range of Motion of stance phase
SWmax: Maximum of swing phase
SWmin: Minimum of swing phase
SWROM: Range of Motion of swing phase
T: Trotting tripedally
TD: touch-down
