# Supplementary material for: Kinematic adaptions to induced short-term pelvic limb lameness in trotting dogs
Source: BMC Vet Res. 2018 Jun 13;14:183. doi: 10.1186/s12917-018-1484-2 (PMC5998594; doi:10.1186/s12917-018-1484-2)
Supplement: Supplementary file 1 — Kinematic results for the thoracic limbs. Mean ± standard deviation (Mean ± SD in °) of the limb, segment and joint angles for all dogs. Kinematic values for the limbs are: angle at touch-down (TD), lift-off (LO) and mid-stance (mid-stance). Kinematic values for the segments and joints are: angle at touch-down (TD) and lift-off (LO) as well as minimum (min), maximum (max) and amplitude (i.e. range of motion, ROM) during stance (ST) and swing (SW) phases. Mean SDs (mSD in °; i.e. SDs from the 10 strides per dog averaged for all dogs) illustrate the relatively low intraindividual variation compared with the interindividual variation (SD of Mean ± SD) and particularly compared with the angular difference between sound and lame trotting (Diff Mean ± SD in °). Note that this mean Diff was calculated by, first, subtracting the lame from the sound values per dog and, second, averaging these angular differences for all dogs (i.e. mean Diff represents the angular changes associated with lame locomotion). Positive Diff values indicate that the angle was greater during sound than lame trotting; negative values indicate the reverse. Significant differences between sound and lame trotting for each limb (I) as well as significant differences between the angular differences of the two limbs (II) at: * P < 0.05, ** P < 0.01, *** P < 0.001. For definition of angles, see Fig. 1 in [16]. (DOC 349 kb) [file 12917_2018_1484_MOESM1_ESM.doc]

**Additional file 1**

**Kinematic results for the thoracic limbs.** Mean±standard deviation (Mean±SD in °) of the limb, segment and joint angles for all dogs. Kinematic values for the limbs are: angle at touch-down (TD), lift-off (LO) and mid-stance (mid-stance). Kinematic values for the segments and joints are: angle at touch-down (TD) and lift-off (LO) as well as minimum (min), maximum (max) and amplitude (i.e. range of motion, ROM) during stance (ST) and swing (SW) phases. Mean SDs (mSD in °; i.e. SDs from the 10 strides per dog averaged for all dogs) illustrate the relatively low intraindividual variation compared with the interindividual variation (SD of Mean±SD) and particularly compared with the angular difference between sound and lame trotting (Diff Mean±SD in °). Note that this mean Diff was calculated by, first, subtracting the lame from the sound values per dog and, second, averaging these angular differences for all dogs (i.e. mean Diff represents the angular changes associated with lame locomotion). Positive Diff values indicate that the angle was greater during sound than lame trotting; negative values indicate the reverse. Significant differences between sound and lame trotting for each limb (I) as well as significant differences between the angular differences of the two limbs (II) at: * *P*<0.05, ** *P*<0.01, *** *P*<0.001. For definition of angles, see Fig. 1 in [16].

|  |  | **Contralateral thoracic limb** | | | | | | | | | | | | |  | **Ipsilateral thoracic limb** | | | | | | | | | | | | |  |  |
| --- | --- | --- | --- | --- | --- | --- | --- | --- | --- | --- | --- | --- | --- | --- | --- | --- | --- | --- | --- | --- | --- | --- | --- | --- | --- | --- | --- | --- | --- | --- |
|  |  | **Sound** | | | | **Lame** | | | | **Diff** | | | **I** | |  | **Sound** | | | | **Lame** | | | | **Diff** | | | **I** | | **II** | |
|  |  | Mean | ± | SD | mSD | Mean | ± | SD | mSD | Mean | ± | SD | *P* |  |  | Mean | ± | SD | mSD | Mean | ± | SD | mSD | Mean | ± | SD | *P* |  | *P* |  |
|  |  |  |  |  |  |  |  |  |  |  |  |  |  |  |  |  |  |  |  |  |  |  |  |  |  |  |  |  |  |  |
| **Limb** | TD | 14.4 | ± | 4.0 | 1.3 | 14.2 | ± | 4.9 | 1.6 | 0.1 | ± | 2.1 | 0.945 |  |  | 15.1 | ± | 2.9 | 1.5 | 14.6 | ± | 4.2 | 1.7 | 0.4 | ± | 2.0 | 0.641 |  | 0.641 |  |
|  | LO | -27.6 | ± | 3.3 | 1.5 | -26.0 | ± | 5.4 | 1.9 | 1.6 | ± | 2.7 | 0.109 |  |  | -28.1 | ± | 3.4 | 1.4 | -28.1 | ± | 5.4 | 1.4 | 0.1 | ± | 2.5 | 0.945 |  | 0.039 | * |
|  | mid-stance | -12.9 | ± | 4.3 | 1.2 | -14.1 | ± | 5.6 | 1.3 | -1.2 | ± | 1.9 | 0.195 |  |  | -12.2 | ± | 3.6 | 1.3 | -11.9 | ± | 5.6 | 1.1 | 0.3 | ± | 2.3 | 0.742 |  | 0.023 | * |
|  |  |  |  |  |  |  |  |  |  |  |  |  |  |  |  |  |  |  |  |  |  |  |  |  |  |  |  |  |  |  |
| **Scapula** | TD | 66.1 | ± | 5.0 | 2.5 | 64.8 | ± | 8.2 | 2.1 | -1.3 | ± | 4.8 | 0.461 |  |  | 65.8 | ± | 4.9 | 2.6 | 63.1 | ± | 7.6 | 2.2 | -2.8 | ± | 4.2 | 0.109 |  | 0.250 |  |
|  | LO | 84.1 | ± | 4.1 | 2.5 | 80.5 | ± | 9.7 | 1.9 | -3.6 | ± | 7.0 | 0.250 |  |  | 84.4 | ± | 4.2 | 2.6 | 83.6 | ± | 8.7 | 2.1 | -0.8 | ± | 5.7 | 0.844 |  | 0.008 | ** |
|  | STmin | 66.1 | ± | 4.1 | 1.9 | 64.8 | ± | 7.9 | 1.6 | -1.3 | ± | 4.8 | 0.461 |  |  | 65.8 | ± | 4.1 | 2.1 | 63.1 | ± | 7.6 | 1.5 | -2.8 | ± | 4.2 | 0.109 |  | 0.250 |  |
|  | STmax | 87.3 | ± | 5.4 | 3.1 | 87.4 | ± | 9.9 | 2.8 | 0.4 | ± | 6.4 | 1.055 |  |  | 86.4 | ± | 5.1 | 3.2 | 84.1 | ± | 10.5 | 3.0 | -2.0 | ± | 6.2 | 0.461 |  | 0.039 | * |
|  | STROM | 21.2 | ± | 1.3 | 1.2 | 22.6 | ± | 2.0 | 1.2 | 1.6 | ± | 2.7 | 0.195 |  |  | 20.6 | ± | 0.9 | 1.1 | 21.0 | ± | 2.9 | 1.5 | 0.8 | ± | 2.6 | 0.383 |  | 0.547 |  |
|  | SWmin | 63.2 | ± | 4.0 | 1.8 | 61.6 | ± | 8.5 | 1.4 | -1.6 | ± | 5.6 | 0.461 |  |  | 63.1 | ± | 4.2 | 2.1 | 61.4 | ± | 8.0 | 1.6 | -1.4 | ± | 4.2 | 0.383 |  | 0.844 |  |
|  | SWmax | 83.7 | ± | 6.3 | 3.1 | 80.0 | ± | 10.7 | 2.7 | -3.6 | ± | 6.6 | 0.250 |  |  | 84.0 | ± | 6.1 | 3.1 | 83.4 | ± | 9.5 | 2.7 | -0.5 | ± | 5.7 | 0.844 |  | 0.008 | ** |
|  | SWROM | 20.4 | ± | 2.3 | 1.3 | 18.4 | ± | 2.2 | 1.3 | -2.1 | ± | 2.7 | 0.109 |  |  | 20.9 | ± | 1.9 | 1.0 | 22.1 | ± | 1.6 | 1.1 | 0.9 | ± | 2.5 | 0.461 |  | 0.055 |  |
|  |  |  |  |  |  |  |  |  |  |  |  |  |  |  |  |  |  |  |  |  |  |  |  |  |  |  |  |  |  |  |
| **Humerus** | TD | 40.1 | ± | 5.5 | 2.3 | 42.4 | ± | 7.4 | 2.6 | 2.3 | ± | 4.5 | 0.195 |  |  | 49.3 | ± | 6.3 | 3.1 | 48.2 | ± | 8.1 | 2.3 | -1.1 | ± | 4.5 | 0.742 |  | 0.008 | ** |
|  | LO | 9.1 | ± | 4.3 | 1.8 | 11.7 | ± | 6.5 | 2.1 | 2.6 | ± | 4.6 | 0.195 |  |  | 14.2 | ± | 5.9 | 2.6 | 14.2 | ± | 9.1 | 2.7 | 0.0 | ± | 5.5 | 0.313 |  | 0.055 |  |
|  | STmin | 9.1 | ± | 4.1 | 1.2 | 11.4 | ± | 5.4 | 1.2 | 1.9 | ± | 4.2 | 0.250 |  |  | 14.2 | ± | 5.6 | 1.1 | 14.2 | ± | 7.7 | 1.2 | 0.0 | ± | 5.5 | 0.313 |  | 0.148 |  |
|  | STmax | 40.1 | ± | 5.5 | 2.6 | 42.4 | ± | 7.4 | 2.7 | 2.3 | ± | 4.5 | 0.195 |  |  | 49.3 | ± | 6.5 | 3.8 | 48.2 | ± | 9.6 | 3.0 | -1.1 | ± | 4.5 | 0.742 |  | 0.008 | ** |
|  | STROM | 30.9 | ± | 1.3 | 1.4 | 31.0 | ± | 2.1 | 1.6 | 0.4 | ± | 2.8 | 0.641 |  |  | 35.1 | ± | 0.9 | 2.7 | 34.0 | ± | 2.0 | 1.8 | -1.2 | ± | 2.7 | 0.195 |  | 0.250 |  |
|  | SWmin | 9.3 | ± | 3.6 | 1.4 | 12.1 | ± | 6.5 | 1.9 | 2.9 | ± | 4.7 | 0.195 |  |  | 14.5 | ± | 6.0 | 1.6 | 14.3 | ± | 8.6 | 1.5 | -0.6 | ± | 5.7 | 0.742 |  | 0.023 | * |
|  | SWmax | 47.0 | ± | 8.2 | 3.6 | 48.0 | ± | 12.3 | 4.2 | 1.1 | ± | 4.1 | 0.250 |  |  | 56.0 | ± | 8.7 | 4.0 | 54.88 | ± | 12.3 | 4.4 | -1.2 | ± | 5.5 | 0.844 |  | 0.078 |  |
|  | SWROM | 37.6 | ± | 4.6 | 2.2 | 35.9 | ± | 5.8 | 2.4 | -1.8 | ± | 3.4 | 0.195 |  |  | 41.5 | ± | 2.7 | 2.4 | 40.5 | ± | 3.6 | 2.9 | -0.6 | ± | 3.2 | 0.641 |  | 0.641 |  |
|  |  |  |  |  |  |  |  |  |  |  |  |  |  |  |  |  |  |  |  |  |  |  |  |  |  |  |  |  |  |  |
| **Antebrachium** | TD | 62.8 | ± | 4.9 | 1.6 | 63.6 | ± | 4.4 | 1.7 | 0.8 | ± | 3.1 | 0.945 |  |  | 64.0 | ± | 3.9 | 1.5 | 66.5 | ± | 4.6 | 1.6 | 2.4 | ± | 2.4 | 0.016 | * | 0.078 |  |
|  | LO | 104.0 | ± | 5.6 | 3.1 | 99.7 | ± | 5.2 | 4.2 | -4.3 | ± | 1.7 | 0.008 | ** |  | 107.2 | ± | 6.1 | 2.6 | 108.0 | ± | 5.9 | 3.3 | 0.9 | ± | 3.6 | 0.313 |  | 0.016 | * |
|  | STmin | 62.8 | ± | 2.6 | 1.0 | 63.6 | ± | 2.5 | 1.0 | 0.8 | ± | 3.0 | 0.945 |  |  | 64.0 | ± | 2.1 | 0.9 | 66.5 | ± | 3.4 | 0.9 | 2.4 | ± | 2.4 | 0.016 | * | 0.078 |  |
|  | STmax | 115.0 | ± | 5.6 | 3.4 | 116.5 | ± | 5.2 | 4.6 | 1.4 | ± | 1.4 | 0.023 | * |  | 117.3 | ± | 6.1 | 2.8 | 118.2 | ± | 5.9 | 3.6 | 0.9 | ± | 4.0 | 0.195 |  | 0.742 |  |
|  | STROM | 52.3 | ± | 3.0 | 2.4 | 52.9 | ± | 2.6 | 3.6 | 0.7 | ± | 3.8 | 0.641 |  |  | 53.2 | ± | 4.0 | 1.9 | 51.7 | ± | 2.5 | 2.7 | -1.5 | ± | 5.3 | 0.742 |  | 0.055 |  |
|  | SWmin | 35.7 | ± | 3.9 | 1.4 | 35.6 | ± | 4.4 | 1.2 | -0.3 | ± | 2.9 | 0.844 |  |  | 38.5 | ± | 2.9 | 1.1 | 43.4 | ± | 4.3 | 1.3 | 4.5 | ± | 5.7 | 0.008 | ** | 0.008 | ** |
|  | SWmax | 102.0 | ± | 9.4 | 4.4 | 97.3 | ± | 9.5 | 5.4 | -4.6 | ± | 2.2 | 0.008 | ** |  | 105.7 | ± | 10.7 | 4.5 | 106.3 | ± | 9.5 | 5.7 | 0.6 | ± | 3.4 | 0.469 |  | 0.016 | * |
|  | SWROM | 66.3 | ± | 5.5 | 3.0 | 61.7 | ± | 5.11 | 4.3 | -4.3 | ± | 3.3 | 0.008 | ** |  | 67.2 | ± | 7.8 | 3.4 | 62.9 | ± | 5.2 | 4.4 | -4.0 | ± | 4.3 | 0.039 | * | 0.641 |  |
|  |  |  |  |  |  |  |  |  |  |  |  |  |  |  |  |  |  |  |  |  |  |  |  |  |  |  |  |  |  |  |
| **Manus** | TD | 132.2 | ± | 11.0 | 3.1 | 129.3 | ± | 9.9 | 3.1 | -2.9 | ± | 3.0 | 0.039 | * |  | 141.9 | ± | 6.7 | 2.8 | 141.8 | ± | 6.9 | 2.7 | -0.2 | ± | 3.0 | 0.742 |  | 0.250 |  |
|  | LO | 15.4 | ± | 16.2 | 8.3 | 2.9 | ± | 13.2 | 7.9 | -12.5 | ± | 3.2 | 0.008 | ** |  | 19.4 | ± | 16.4 | 7.1 | 21.6 | ± | 15.1 | 7.9 | 2.2 | ± | 7.7 | 0.461 |  | 0.008 | ** |
|  | STmin | 15.4 | ± | 9.5 | 1.9 | 2.9 | ± | 9.8 | 1.3 | -12.5 | ± | 3.2 | 0.008 | ** |  | 19.4 | ± | 6.1 | 1.3 | 21.6 | ± | 5.2 | 1.4 | 2.2 | ± | 7.7 | 0.461 |  | 0.008 | ** |
|  | STmax | 132.3 | ± | 16.2 | 9.4 | 129.3 | ± | 13.5 | 10.2 | -2.6 | ± | 3.3 | 0.078 |  |  | 141.9 | ± | 16.4 | 8.6 | 141.8 | ± | 15.1 | 9.1 | -0.4 | ± | 3.0 | 0.945 |  | 0.109 |  |
|  | STROM | 116.9 | ± | 6.7 | 7.5 | 126.4 | ± | 3.7 | 8.9 | 9.9 | ± | 5.0 | 0.016 | * |  | 122.6 | ± | 10.3 | 7.3 | 120.2 | ± | 9.8 | 7.7 | -2.6 | ± | 9.4 | 0.383 |  | 0.0156 | * |
|  | SWmin | -4.5 | ± | 8.1 | 2.0 | -11.6 | ± | 6.0 | 1.5 | -5.5 | ± | 4.4 | 0.016 | * |  | -0.5 | ± | 5.9 | 1.5 | 4.0 | ± | 5.3 | 1.6 | 5.8 | ± | 7.5 | 0.078 |  | 0.008 | ** |
|  | SWmax | 136.8 | ± | 15.6 | 9.4 | 134.9 | ± | 16.6 | 9.6 | -1.1 | ± | 3.0 | 0.461 |  |  | 143.2 | ± | 16.3 | 8.4 | 142.2 | ± | 17.4 | 9.5 | -0.4 | ± | 5.0 | 1.055 |  | 0.641 |  |
|  | SWROM | 141.2 | ± | 7.5 | 7.4 | 146.5 | ± | 10.6 | 8.1 | 4.4 | ± | 5.8 | 0.055 |  |  | 143.7 | ± | 10.4 | 6.9 | 138.2 | ± | 12.0 | 7.9 | -6.2 | ± | 10.2 | 0.148 |  | 0.008 | ** |
|  |  |  |  |  |  |  |  |  |  |  |  |  |  |  |  |  |  |  |  |  |  |  |  |  |  |  |  |  |  |  |
| **Shoulder joint** | TD | 106.1 | ± | 6.4 | 2.1 | 107.2 | ± | 8.6 | 2.0 | 0.9 | ± | 4.7 | 0.945 |  |  | 115.1 | ± | 9.2 | 3.2 | 111.2 | ± | 9.2 | 2.1 | -3.9 | ± | 4.2 | 0.055 |  | 0.039 | * |
|  | LO | 93.2 | ± | 3.4 | 1.6 | 92.2 | ± | 8.0 | 2.0 | -1.0 | ± | 6.9 | 0.313 |  |  | 98.6 | ± | 6.2 | 2.6 | 97.8 | ± | 7.9 | 2.5 | -0.8 | ± | 5.2 | 0.742 |  | 0.844 |  |
|  | STmin | 93.2 | ± | 3.3 | 1.3 | 92.2 | ± | 7.7 | 1.2 | -1.1 | ± | 6.9 | 0.250 |  |  | 98.6 | ± | 4.9 | 1.5 | 97.8 | ± | 7.6 | 1.3 | -1.0 | ± | 5.1 | 0.383 |  | 0.945 |  |
|  | STmax | 106.1 | ± | 12.9 | 2.8 | 107.2 | ± | 9.6 | 2.8 | 1.1 | ± | 4.7 | 0.945 |  |  | 115.1 | ± | 9.2 | 4.2 | 111.2 | ± | 9.2 | 2.9 | -3.9 | ± | 4.2 | 0.055 |  | 0.039 | * |
|  | STROM | 12.9 | ± | 3.1 | 1.5 | 15.0 | ± | 2.0 | 1.6 | 2.2 | ± | 4.1 | 0.195 |  |  | 16.6 | ± | 4.3 | 2.7 | 13.4 | ± | 1.6 | 1.6 | -2.8 | ± | 3.1 | 0.078 |  | 0.023 | * |
|  | SWmin | 90.7 | ± | 3.4 | 1.2 | 90.1 | ± | 7.2 | 1.2 | -0.8 | ± | 5.5 | 0.641 |  |  | 93.7 | ± | 4.6 | 1.5 | 92.6 | ± | 7.1 | 1.4 | -1.1 | ± | 4.0 | 0.469 |  | 0.742 |  |
|  | SWmax | 110.2 | ± | 8.0 | 3.3 | 109.6 | ± | 9.1 | 3.5 | -0.4 | ± | 3.3 | 0.461 |  |  | 119.1 | ± | 10.0 | 4.3 | 116.3 | ± | 10.5 | 3.9 | -3.0 | ± | 5.1 | 0.250 |  | 0.148 |  |
|  | SWROM | 19.5 | ± | 4.6 | 2.2 | 19.5 | ± | 1.9 | 2.2 | 0.4 | ± | 3.6 | 0.844 |  |  | 25.4 | ± | 5.3 | 2.9 | 23.7 | ± | 3.4 | 2.5 | -1.9 | ± | 4.0 | 0.383 |  | 0.547 |  |
|  |  |  |  |  |  |  |  |  |  |  |  |  |  |  |  |  |  |  |  |  |  |  |  |  |  |  |  |  |  |  |
| **Elbow joint** | TD | 102.8 | ± | 7.8 | 2.6 | 105.9 | ± | 10.6 | 2.7 | 3.1 | ± | 6.7 | 0.148 |  |  | 113.3 | ± | 8.3 | 3.2 | 114.6 | ± | 11.0 | 2.1 | 1.3 | ± | 6.0 | 0.461 |  | 0.742 |  |
|  | LO | 113.1 | ± | 8.7 | 3.6 | 111.4 | ± | 8.1 | 5.0 | -1.7 | ± | 4.3 | 0.461 |  |  | 121.3 | ± | 7.5 | 4.1 | 122.2 | ± | 9.4 | 5.1 | 0.9 | ± | 7.1 | 1.000 |  | 0.313 |  |
|  | STmin | 96.6 | ± | 5.4 | 1.6 | 99.7 | ± | 5.6 | 1.4 | 3.3 | ± | 6.1 | 0.156 |  |  | 107.1 | ± | 4.7 | 1.7 | 107.9 | ± | 6.9 | 1.5 | 0.9 | ± | 5.8 | 0.742 |  | 0.195 |  |
|  | STmax | 125.3 | ± | 8.7 | 4.0 | 129.1 | ± | 10.6 | 5.5 | 3.7 | ± | 4.6 | 0.078 |  |  | 134.1 | ± | 8.3 | 4.9 | 135.4 | ± | 11.0 | 5.3 | 1.3 | ± | 6.7 | 0.547 |  | 0.641 |  |
|  | STROM | 28.8 | ± | 3.2 | 2.3 | 29.4 | ± | 5.0 | 4.1 | 0.4 | ± | 3.5 | 0.742 |  |  | 26.9 | ± | 3.7 | 3.3 | 27.6 | ± | 4.1 | 3.7 | 0.4 | ± | 5.4 | 0.461 |  | 0.742 |  |
|  | SWmin | 59.5 | ± | 7.6 | 1.9 | 60.1 | ± | 8.2 | 1.8 | 0.4 | ± | 4.7 | 0.641 |  |  | 68.2 | ± | 7.4 | 1.7 | 70.5 | ± | 9.6 | 1.8 | 2.1 | ± | 7.1 | 0.461 |  | 0.313 |  |
|  | SWmax | 111.3 | ± | 11.0 | 5.0 | 109.4 | ± | 16.7 | 6.4 | -1.5 | ± | 4.3 | 0.547 |  |  | 120.2 | ± | 12.3 | 5.8 | 120.6 | ± | 15.9 | 6.8 | 0.7 | ± | 7.2 | 0.844 |  | 0.313 |  |
|  | SWROM | 51.8 | ± | 3.5 | 3.1 | 49.3 | ± | 8.5 | 4.6 | -1.9 | ± | 4.4 | 0.742 |  |  | 51.9 | ± | 4.9 | 4.1 | 50.1 | ± | 6.3 | 5.0 | -1.4 | ± | 2.2 | 0.109 |  | 0.742 |  |
|  |  |  |  |  |  |  |  |  |  |  |  |  |  |  |  |  |  |  |  |  |  |  |  |  |  |  |  |  |  |  |
| **Carpal joint** | TD | 195.0 | ± | 11.4 | 3.2 | 192.9 | ± | 8.9 | 2.9 | -2.1 | ± | 3.6 | 0.195 |  |  | 206.0 | ± | 7.1 | 2.6 | 208.2 | ± | 7.4 | 2.6 | 2.3 | ± | 4.0 | 1.148 |  | 0.109 |  |
|  | LO | 120.1 | ± | 19.7 | 11.2 | 102.5 | ± | 17.7 | 12.2 | -17.6 | ± | 3.7 | 0.008 | ** |  | 126.5 | ± | 19.0 | 8.9 | 129.9 | ± | 17.1 | 11.0 | 3.4 | ± | 8.2 | 0.250 |  | 0.008 | ** |
|  | STmin | 120.1 | ± | 8.4 | 1.6 | 102.5 | ± | 8.7 | 0.9 | -17.6 | ± | 3.7 | 0.008 | ** |  | 126.5 | ± | 6.2 | 0.9 | 129.9 | ± | 6.8 | 1.0 | 3.4 | ± | 8.2 | 0.250 |  | 0.008 | ** |
|  | STmax | 207.8 | ± | 19.7 | 12.2 | 205.5 | ± | 17.7 | 14.0 | -1.6 | ± | 3.1 | 0.195 |  |  | 220.3 | ± | 19.0 | 10.7 | 220.4 | ± | 17.4 | 12.1 | 0.1 | ± | 5.2 | 0.641 |  | 1.055 |  |
|  | STROM | 87.7 | ± | 11.2 | 10.6 | 103.0 | ± | 9.0 | 13.0 | 16.0 | ± | 3.8 | 0.008 | ** |  | 93.7 | ± | 12.8 | 9.7 | 90.5 | ± | 10.4 | 11.1 | -3.3 | ± | 7.9 | 0.297 |  | 0.008 | ** |
|  | SWmin | 79.2 | ± | 10.0 | 1.8 | 71.3 | ± | 8.1 | 1.3 | -6.6 | ± | 5.9 | 0.023 | * |  | 87.4 | ± | 6.8 | 1.4 | 93.5 | ± | 6.9 | 1.5 | 6.5 | ± | 7.2 | 0.055 |  | 0.008 | ** |
|  | SWmax | 193.9 | ± | 20.5 | 11.1 | 191.3 | ± | 18.5 | 11.0 | -2.5 | ± | 2.5 | 0.039 | * |  | 204.2 | ± | 19.2 | 9.3 | 205.6 | ± | 17.2 | 11.7 | 1.3 | ± | 4.7 | 0.945 |  | 0.078 |  |
|  | SWROM | 114.7 | ± | 10.4 | 9.4 | 120.0 | ± | 10.4 | 9.7 | 4.1 | ± | 5.6 | 0.109 |  |  | 116.8 | ± | 12.4 | 7.9 | 112.1 | ± | 10.3 | 10.1 | -5.2 | ± | 8.5 | 0.148 |  | 0.023 | * |
|  |  |  |  |  |  |  |  |  |  |  |  |  |  |  |  |  |  |  |  |  |  |  |  |  |  |  |  |  |  |  |
