# Supplementary material for: Kinematic adaptions to induced short-term pelvic limb lameness in trotting dogs
Source: BMC Vet Res. 2018 Jun 13;14:183. doi: 10.1186/s12917-018-1484-2 (PMC5998594; doi:10.1186/s12917-018-1484-2)
Supplement: Supplementary file 2 — Kinematic results for the pelvic limbs. For further explanation, see Additional file 1. (DOC 332 kb) [file 12917_2018_1484_MOESM2_ESM.doc]

**Additional file 2**

**Kinematic results for the pelvic limbs.** For further explanation, see Additional file 1.

|  |  | **Contralateral pelvic limb** | | | | | | | | | | | | |  | **Ipsilateral pelvic limb** | | | | | | | | | | | | |  |  |
| --- | --- | --- | --- | --- | --- | --- | --- | --- | --- | --- | --- | --- | --- | --- | --- | --- | --- | --- | --- | --- | --- | --- | --- | --- | --- | --- | --- | --- | --- | --- |
|  |  | **Sound** | | | | **Lame** | | | | **Diff** | | | **I** | |  | **Sound** | | | | **Lame** | | | | **Diff** | | | **I** | | **II** | |
|  |  | Mean | ± | SD | mSD | Mean | ± | SD | mSD | Mean | ± | SD | *P* |  |  | Mean | ± | SD | mSD | Mean | ± | SD | mSD | Mean | ± | SD | *P* |  | *P* |  |
|  |  |  |  |  |  |  |  |  |  |  |  |  |  |  |  |  |  |  |  |  |  |  |  |  |  |  |  |  |  |  |
| **Limb** | TD | 15.3 | ± | 2.6 | 2.0 | 20.1 | ± | 4.2 | 1.7 | 4.8 | ± | 3.4 | 0.016 | * |  | 13.6 | ± | 3.2 | 1.6 | 14.1 | ± | 4.2 | 2.3 | 0.5 | ± | 2.9 | 0.938 |  | 0.078 |  |
|  | LO | -25.8 | ± | 3.5 | 0.9 | -24.7 | ± | 3.1 | 1.0 | 1.1 | ± | 2.0 | 0.219 |  |  | -26.4 | ± | 2.6 | 1.1 | -30.2 | ± | 3.2 | 1.4 | -3.8 | ± | 2.5 | 0.016 | * | 0.031 | * |
|  | mid-stance | -7.5 | ± | 2.9 | 1.1 | -4.4 | ± | 3.4 | 1.1 | 3.1 | ± | 2.4 | 0.016 | * |  | -8.9 | ± | 2.5 | 1.2 | -9.7 | ± | 2.8 | 1.8 | -0.8 | ± | 2.4 | 0.297 |  | 0.047 | * |
|  |  |  |  |  |  |  |  |  |  |  |  |  |  |  |  |  |  |  |  |  |  |  |  |  |  |  |  |  |  |  |
| **Pelvis** | TD | 150.4 | ± | 5.5 | 1.1 | 146.6 | ± | 6.1 | 1.1 | -3.8 | ± | 3.5 | 0.023 | * |  | 151.1 | ± | 4.2 | 1.1 | 149.3 | ± | 5.2 | 1.3 | -1.7 | ± | 2.8 | 0.250 |  | 0.313 |  |
|  | LO | 152.6 | ± | 4.6 | 0.8 | 150.8 | ± | 4.9 | 1.0 | -1.9 | ± | 2.2 | 0.078 |  |  | 152.5 | ± | 3.7 | 0.8 | 150.2 | ± | 3.9 | 0.9 | -2.3 | ± | 2.7 | 0.078 |  | 0.547 |  |
|  | STmin | 150.4 | ± | 4.6 | 0.6 | 146.6 | ± | 4.7 | 0.7 | -4.3 | ± | 3.3 | 0.016 | * |  | 151.1 | ± | 3.7 | 0.6 | 149.3 | ± | 3.7 | 0.7 | -2.8 | ± | 2.6 | 0.039 | * | 0.109 |  |
|  | STmax | 157.6 | ± | 5.5 | 1.3 | 154.4 | ± | 6.1 | 1.7 | -3.1 | ± | 2.5 | 0.016 | * |  | 157.1 | ± | 5.0 | 1.3 | 155.3 | ± | 5.2 | 1.7 | -1.7 | ± | 2.7 | 0.109 |  | 0.023 | * |
|  | STROM | 7.2 | ± | 1.0 | 0.7 | 7.8 | ± | 1.4 | 1.0 | 1.2 | ± | 1.4 | 0.109 |  |  | 6.0 | ± | 1.3 | 0.7 | 6.0 | ± | 1.6 | 1.0 | 1.1 | ± | 1.2 | 0.039 | * | 0.844 |  |
|  | SWmin | 149.2 | ± | 4.5 | 0.5 | 146.0 | ± | 4.9 | 0.6 | -3.6 | ± | 3.3 | 0.055 |  |  | 149.7 | ± | 3.5 | 0.6 | 147.9 | ± | 3.9 | 0.7 | -2.3 | ± | 2.6 | 0.078 |  | 0.383 |  |
|  | SWmax | 156.0 | ± | 6.4 | 1.4 | 154.6 | ± | 6.0 | 1.6 | -1.7 | ± | 2.3 | 0.078 |  |  | 156.7 | ± | 4.7 | 1.3 | 153.3 | ± | 5.1 | 1.6 | -3.4 | ± | 2.5 | 0.016 | * | 0.008 | ** |
|  | SWROM | 6.7 | ± | 1.8 | 0.8 | 8.6 | ± | 1.1 | 1.0 | 1.9 | ± | 2.1 | 0.039 | * |  | 7.0 | ± | 1.2 | 0.7 | 5.4 | ± | 1.2 | 0.9 | -1.1 | ± | 1.7 | 0.195 |  | 0.039 | * |
|  |  |  |  |  |  |  |  |  |  |  |  |  |  |  |  |  |  |  |  |  |  |  |  |  |  |  |  |  |  |  |
| **Femur** | TD | 59.0 | ± | 5.7 | 1.6 | 54.1 | ± | 7.1 | 1.5 | -4.9 | ± | 2.8 | 0.008 | ** |  | 61.5 | ± | 6.7 | 1.4 | 58.7 | ± | 5.7 | 1.7 | -2.8 | ± | 3.0 | 0.039 | * | 0.250 |  |
|  | LO | 83.0 | ± | 5.4 | 0.8 | 81.1 | ± | 6.1 | 1.0 | -1.9 | ± | 2.1 | 0.063 |  |  | 85.1 | ± | 6.6 | 0.9 | 84.4 | ± | 7.3 | 1.2 | -0.6 | ± | 2.5 | 0.547 |  | 0.250 |  |
|  | STmin | 59.0 | ± | 4.9 | 0.7 | 54.1 | ± | 6.1 | 0.7 | -4.9 | ± | 2.8 | 0.008 | ** |  | 61.5 | ± | 6.4 | 0.7 | 58.7 | ± | 5.6 | 1.0 | -2.8 | ± | 3.1 | 0.039 | * | 0.250 |  |
|  | STmax | 83.0 | ± | 5.9 | 1.9 | 81.1 | ± | 7.7 | 1.9 | -1.8 | ± | 2.1 | 0.063 |  |  | 85.1 | ± | 7.8 | 1.6 | 84.4 | ± | 7.3 | 2.5 | -0.5 | ± | 2.4 | 0.547 |  | 0.195 |  |
|  | STROM | 24.1 | ± | 1.0 | 1.2 | 27.0 | ± | 1.5 | 1.3 | 3.0 | ± | 3.1 | 0.039 | * |  | 23.5 | ± | 1.4 | 0.9 | 25.7 | ± | 1.7 | 1.5 | 2.3 | ± | 2.3 | 0.039 | * | 0.844 |  |
|  | SWmin | 54.7 | ± | 5.4 | 0.7 | 50.7 | ± | 6.1 | 0.8 | -3.9 | ± | 2.2 | 0.008 | ** |  | 57.4 | ± | 6.1 | 0.7 | 53.9 | ± | 5.7 | 1.0 | -3.6 | ± | 2.9 | 0.008 | ** | 0.945 |  |
|  | SWmax | 83.3 | ± | 6.9 | 1.8 | 81.1 | ± | 7.6 | 2.0 | -2.1 | ± | 2.1 | 0.008 | ** |  | 85.5 | ± | 7.7 | 1.7 | 84.5 | ± | 10.1 | 2.3 | -1.0 | ± | 2.2 | 0.313 |  | 0.195 |  |
|  | SWROM | 28.6 | ± | 1.5 | 1.2 | 30.4 | ± | 1.5 | 1.2 | 1.7 | ± | 3.5 | 0.313 |  |  | 28.1 | ± | 1.6 | 1.0 | 30.6 | ± | 4.4 | 1.3 | 2.6 | ± | 1.9 | 0.016 | * | 0.547 |  |
|  |  |  |  |  |  |  |  |  |  |  |  |  |  |  |  |  |  |  |  |  |  |  |  |  |  |  |  |  |  |  |
| **Crus** | TD | 80.1 | ± | 4.4 | 2.9 | 84.0 | ± | 5.2 | 2.8 | 3.8 | ± | 3.7 | 0.039 | * |  | 79.3 | ± | 4.4 | 2.3 | 80.2 | ± | 6.9 | 4.0 | 0.9 | ± | 5.3 | 0.742 |  | 0.383 |  |
|  | LO | 39.9 | ± | 3.1 | 1.2 | 40.1 | ± | 2.9 | 1.3 | 0.3 | ± | 1.1 | 0.469 |  |  | 40.5 | ± | 5.1 | 1.4 | 34.9 | ± | 4.1 | 1.4 | -5.6 | ± | 2.5 | 0.008 | ** | 0.008 | ** |
|  | STmin | 39.9 | ± | 3.1 | 0.9 | 40.1 | ± | 2.5 | 1.1 | 0.2 | ± | 1.1 | 0.578 |  |  | 40.5 | ± | 3.8 | 1.1 | 34.9 | ± | 3.8 | 1.3 | -5.6 | ± | 2.5 | 0.008 | ** | 0.008 | ** |
|  | STmax | 80.1 | ± | 4.4 | 3.0 | 84.0 | ± | 5.2 | 3.2 | 3.8 | ± | 3.7 | 0.039 | * |  | 79.3 | ± | 5.3 | 2.4 | 80.2 | ± | 6.9 | 4.0 | 0.9 | ± | 5.3 | 0.742 |  | 0.383 |  |
|  | STROM | 40.3 | ± | 1.3 | 2.1 | 43.8 | ± | 2.6 | 2.1 | 3.6 | ± | 3.0 | 0.039 | * |  | 38.8 | ± | 1.6 | 1.3 | 45.3 | ± | 3.1 | 2.7 | 6.5 | ± | 4.1 | 0.008 | ** | 0.313 |  |
|  | SWmin | 23.8 | ± | 3.2 | 1.0 | 29.7 | ± | 3.0 | 1.1 | 5.4 | ± | 2.6 | 0.008 | ** |  | 22.9 | ± | 2.5 | 0.9 | 19.2 | ± | 1.9 | 1.3 | -4.1 | ± | 2.5 | 0.016 | * | 0.008 | ** |
|  | SWmax | 89.4 | ± | 7.9 | 5.0 | 92.3 | ± | 8.8 | 4.8 | 3.0 | ± | 2.7 | 0.023 | * |  | 89.1 | ± | 5.7 | 3.8 | 89.3 | ± | 9.9 | 5.7 | 0.9 | ± | 3.1 | 0.742 |  | 0.250 |  |
|  | SWROM | 65.5 | ± | 4.7 | 4.0 | 62.6 | ± | 5.8 | 3.7 | -2.4 | ± | 3.8 | 0.148 |  |  | 66.2 | ± | 3.2 | 3.0 | 70.1 | ± | 8.0 | 4.5 | 5.0 | ± | 4.3 | 0.023 | * | 0.039 | * |
|  |  |  |  |  |  |  |  |  |  |  |  |  |  |  |  |  |  |  |  |  |  |  |  |  |  |  |  |  |  |  |
| **Pes** | TD | 54.8 | ± | 3.2 | 2.1 | 46.6 | ± | 5.7 | 2.8 | -8.2 | ± | 6.2 | 0.008 | ** |  | 56.3 | ± | 6.3 | 1.9 | 62.5 | ± | 6.6 | 2.4 | 6.2 | ± | 5.4 | 0.016 | * | 0.008 | ** |
|  | LO | 116.2 | ± | 6.2 | 1.9 | 114.8 | ± | 4.6 | 2.1 | -1.4 | ± | 5.4 | 0.688 |  |  | 115.9 | ± | 4.8 | 1.7 | 123.2 | ± | 4.7 | 2.7 | 7.3 | ± | 5.9 | 0.461 |  | 0.023 | * |
|  | STmin | 53.4 | ± | 3.2 | 1.4 | 43.9 | ± | 4.6 | 1.6 | -9.4 | ± | 6.5 | 0.008 | ** |  | 55.6 | ± | 4.8 | 1.4 | 62.5 | ± | 4.7 | 1.7 | 7.4 | ± | 5.0 | 0.016 | * | 0.008 | ** |
|  | STmax | 116.2 | ± | 6.2 | 3.2 | 114.8 | ± | 6.8 | 3.3 | -1.2 | ± | 5.0 | 0.688 |  |  | 115.9 | ± | 6.8 | 2.9 | 123.2 | ± | 7.0 | 3.4 | 7.3 | ± | 5.9 | 0.383 |  | 0.023 | * |
|  | STROM | 62.8 | ± | 3.0 | 1.8 | 70.9 | ± | 2.2 | 1.7 | 8.3 | ± | 4.9 | 0.008 | ** |  | 60.3 | ± | 2.0 | 1.6 | 60.7 | ± | 2.3 | 1.7 | -0.1 | ± | 5.1 | 1.055 |  | 0.008 | ** |
|  | SWmin | 52.4 | ± | 2.5 | 1.0 | 47.6 | ± | 4.4 | 1.0 | -4.9 | ± | 3.8 | 0.008 | ** |  | 53.7 | ± | 3.9 | 0.8 | 54.4 | ± | 3.3 | 1.4 | 0.1 | ± | 5.2 | 0.945 |  | 0.023 | * |
|  | SWmax | 117.5 | ± | 6.6 | 4.0 | 115.0 | ± | 8.5 | 3.9 | -2.5 | ± | 5.2 | 0.383 |  |  | 117.7 | ± | 11.9 | 3.4 | 127.6 | ± | 14.1 | 4.7 | 10.3 | ± | 3.7 | 0.008 | ** | 0.008 | ** |
|  | SWROM | 65.2 | ± | 4.1 | 3.0 | 67.4 | ± | 4.1 | 2.9 | 2.5 | ± | 4.4 | 0.313 |  |  | 64.1 | ± | 8.0 | 2.6 | 73.2 | ± | 10.9 | 3.3 | 10.2 | ± | 4.0 | 0.008 | ** | 0.008 | ** |
|  |  |  |  |  |  |  |  |  |  |  |  |  |  |  |  |  |  |  |  |  |  |  |  |  |  |  |  |  |  |  |
| **Hip joint** | TD | 97.6 | ± | 13.4 | 1.4 | 96.7 | ± | 12.9 | 1.7 | -0.9 | ± | 2.6 | 0.219 |  |  | 97.5 | ± | 9.8 | 1.4 | 96.3 | ± | 9.1 | 1.9 | -1.2 | ± | 2.5 | 0.148 |  | 0.547 |  |
|  | LO | 117.0 | ± | 13.4 | 1.3 | 116.9 | ± | 13.7 | 1.3 | -0.1 | ± | 2.9 | 1.055 |  |  | 116.8 | ± | 11.5 | 1.0 | 119.9 | ± | 11.6 | 1.4 | 3.1 | ± | 1.6 | 0.008 | ** | 0.055 |  |
|  | STmin | 97.6 | ± | 13.4 | 0.7 | 96.7 | ± | 12.9 | 1.1 | -0.9 | ± | 2.6 | 0.213 |  |  | 97.5 | ± | 9.8 | 0.7 | 96.3 | ± | 9.1 | 1.1 | -1.2 | ± | 2.5 | 0.148 |  | 0.547 |  |
|  | STmax | 117.0 | ± | 14.3 | 1.8 | 116.9 | ± | 14.9 | 2.2 | 0.1 | ± | 2.8 | 1.055 |  |  | 116.8 | ± | 12.3 | 1.7 | 119.9 | ± | 11.8 | 2.6 | 3.3 | ± | 1.6 | 0.008 | ** | 0.055 |  |
|  | STROM | 19.3 | ± | 0.9 | 1.1 | 20.1 | ± | 2.0 | 1.1 | 1.0 | ± | 2.5 | 0.461 |  |  | 19.3 | ± | 2.5 | 1.1 | 23.6 | ± | 2.7 | 1.5 | 4.5 | ± | 2.8 | 0.008 | ** | 0.078 |  |
|  | SWmin | 94.5 | ± | 11.9 | 0.6 | 92.3 | ± | 12.3 | 0.9 | -1.8 | ± | 2.6 | 0.078 |  |  | 95.3 | ± | 9.0 | 0.7 | 95.0 | ± | 9.0 | 1.0 | -1.8 | ± | 2.8 | 0.055 |  | 0.844 |  |
|  | SWmax | 117.3 | ± | 14.5 | 2.1 | 116.8 | ± | 14.1 | 2.2 | -0.7 | ± | 2.9 | 0.547 |  |  | 117.3 | ± | 12.3 | 1.9 | 120.0 | ± | 13.6 | 2.2 | 2.5 | ± | 1.8 | 0.008 | ** | 0.039 | * |
|  | SWROM | 22.8 | ± | 2.6 | 1.5 | 24.5 | ± | 1.7 | 1.3 | 1.0 | ± | 2.9 | 0.313 |  |  | 22.1 | ± | 2.8 | 1.2 | 25.0 | ± | 4.55 | 1.3 | 4.3 | ± | 3.1 | 0.008 | ** | 0.078 |  |
|  |  |  |  |  |  |  |  |  |  |  |  |  |  |  |  |  |  |  |  |  |  |  |  |  |  |  |  |  |  |  |
| **Knee joint** | TD | 139.1 | ± | 7.6 | 2.0 | 138.1 | ± | 8.9 | 2.1 | -1.0 | ± | 2.4 | 0.250 |  |  | 140.8 | ± | 6.9 | 1.5 | 138.9 | ± | 8.5 | 3.1 | -2.0 | ± | 3.0 | 0.008 |  | 0.547 |  |
|  | LO | 122.9 | ± | 5.6 | 1.3 | 121.3 | ± | 6.8 | 1.6 | -1.7 | ± | 2.2 | 0.109 |  |  | 125.6 | ± | 7.9 | 1.5 | 119.3 | ± | 9.2 | 1.6 | -6.3 | ± | 3.5 | 0.008 | ** | 0.016 | * |
|  | STmin | 121.2 | ± | 5.6 | 0.9 | 116.3 | ± | 6.8 | 1.1 | -4.0 | ± | 2.7 | 0.016 | * |  | 123.2 | ± | 6.9 | 1.0 | 119.3 | ± | 6.7 | 1.2 | -4.0 | ± | 5.0 | 0.055 | ** | 0.844 | * |
|  | STmax | 139.1 | ± | 7.6 | 2.3 | 138.1 | ± | 9.6 | 3.0 | -1.0 | ± | 2.4 | 0.250 |  |  | 140.8 | ± | 9.0 | 2.1 | 138.9 | ± | 9.2 | 3.2 | -1.6 | ± | 2.6 | 0.078 |  | 0.641 |  |
|  | STROM | 17.9 | ± | 2.1 | 1.4 | 21.8 | ± | 2.8 | 1.9 | 2.9 | ± | 2.5 | 0.039 | * |  | 17.6 | ± | 2.1 | 1.1 | 19.6 | ± | 2.5 | 2.0 | 2.6 | ± | 4.4 | 0.195 |  | 0.742 |  |
|  | SWmin | 93.3 | ± | 5.6 | 1.0 | 97.9 | ± | 6.5 | 1.0 | -1.2 | ± | 2.5 | 0.008 | ** |  | 95.8 | ± | 5.2 | 0.8 | 87.2 | ± | 4.3 | 1.3 | -9.3 | ± | 3.5 | 0.008 | ** | 0.008 | ** |
|  | SWmax | 144.8 | ± | 8.7 | 4.6 | 143.6 | ± | 9.1 | 4.4 | -1.2 | ± | 2.5 | 0.250 |  |  | 147.0 | ± | 9.1 | 3.6 | 145.2 | ± | 13.2 | 5.7 | -1.1 | ± | 2.0 | 0.148 |  | 0.844 |  |
|  | SWROM | 51.6 | ± | 3.1 | 3.7 | 45.7 | ± | 2.6 | 3.4 | -5.5 | ± | 3.0 | 0.008 | ** |  | 51.2 | ± | 3.9 | 2.8 | 57.9 | ± | 8.9 | 4.4 | 8.2 | ± | 3.8 | 0.008 | ** | 0.008 | ** |
|  |  |  |  |  |  |  |  |  |  |  |  |  |  |  |  |  |  |  |  |  |  |  |  |  |  |  |  |  |  |  |
| **Tarsal joint** | TD | 134.9 | ± | 2.8 | 3.5 | 130.5 | ± | 7.1 | 4.6 | -4.4 | ± | 5.4 | 0.039 | * |  | 135.6 | ± | 8.0 | 2.7 | 142.7 | ± | 8.5 | 3.2 | 7.1 | ± | 8.6 | 0.078 |  | 0.016 | * |
|  | LO | 156.1 | ± | 5.1 | 1.6 | 155.0 | ± | 5.2 | 2.0 | -1.1 | ± | 5.2 | 0.945 |  |  | 156.4 | ± | 7.2 | 1.5 | 158.1 | ± | 4.0 | 2.2 | 1.7 | ± | 6.4 | 0.547 |  | 0.250 |  |
|  | STmin | 115.7 | ± | 2.2 | 1.3 | 107.2 | ± | 4.9 | 1.6 | -8.5 | ± | 5.1 | 0.008 | ** |  | 117.3 | ± | 6.7 | 1.2 | 131.9 | ± | 4.0 | 1.5 | 14.1 | ± | 7.9 | 0.016 | * | 0.008 | ** |
|  | STmax | 156.1 | ± | 5.1 | 3.7 | 155.0 | ± | 7.1 | 5.2 | -0.7 | ± | 4.3 | 0.945 |  |  | 156.4 | ± | 8.7 | 3.5 | 158.1 | ± | 8.5 | 3.9 | 1.7 | ± | 6.4 | 0.016 | * | 0.461 |  |
|  | STROM | 40.4 | ± | 2.9 | 2.5 | 47.8 | ± | 2.2 | 3.7 | 7.8 | ± | 2.9 | 0.008 | ** |  | 39.1 | ± | 2.0 | 2.3 | 26.2 | ± | 4.5 | 2.4 | -12.4 | ± | 6.2 | 0.008 | ** | 0.008 | ** |
|  | SWmin | 108.7 | ± | 2.2 | 1.1 | 110.1 | ± | 5.3 | 1.1 | 1.5 | ± | 4.0 | 0.250 |  |  | 109.3 | ± | 6.8 | 0.8 | 108.6 | ± | 4.1 | 1.3 | -1.4 | ± | 5.3 | 0.461 |  | 0.844 |  |
|  | SWmax | 156.0 | ± | 6.8 | 4.6 | 154.5 | ± | 10.2 | 4.9 | -1.6 | ± | 5.3 | 0.844 |  |  | 156.7 | ± | 10.5 | 3.8 | 159.5 | ± | 13.1 | 6.4 | 3.2 | ± | 5.6 | 0.313 |  | 0.109 |  |
|  | SWROM | 47.4 | ± | 3.8 | 3.5 | 44.4 | ± | 4.9 | 3.8 | -3.1 | ± | 6.0 | 0.250 |  |  | 47.4 | ± | 3.7 | 3.0 | 50.9 | ± | 9.0 | 5.1 | 4.6 | ± | 3.2 | 0.008 | ** | 0.016 | * |
|  |  |  |  |  |  |  |  |  |  |  |  |  |  |  |  |  |  |  |  |  |  |  |  |  |  |  |  |  |  |  |
